# GLIPR1-ΔTM synergizes with docetaxel in cell death and suppresses resistance to docetaxel in prostate cancer cells

**DOI:** 10.1186/s12943-015-0395-0

**Published:** 2015-06-19

**Authors:** Styliani Karanika, Theodoros Karantanos, Shinji Kurosaka, Jianxiang Wang, Takahiro Hirayama, Guang Yang, Sanghee Park, Alexei A. Golstov, Ryuta Tanimoto, Likun Li, Timothy C. Thompson

**Affiliations:** Department of Genitourinary Medical Oncology – Research, Unit 18-3, The University of Texas MD Anderson Cancer Center, 1515 Holcombe Boulevard, Houston, TX USA; Department of Urology, Kitasato University School of Medicine, Sagamihara, Kanagawa Japan; Department of Thoracic & Cardio Surgery, The University of Texas MD Anderson Cancer Center, Houston, TX USA; Department of Urology, Thomas Jefferson University Hospital, Philadelphia, PA USA

**Keywords:** Prostate cancer, GLIPR1-ΔTM, Docetaxel, JNK, ERK1/2, c-Myc, CXCR4, Combination treatment

## Abstract

**Background:**

Docetaxel is the first chemotherapy agent approved for treatment of metastatic castration-resistant prostate cancer (mCRPC). The limited survival benefit associated with the quick emergence of resistance and systemic toxicity diminished its efficacy. JNK-mediated apoptosis is one of the mechanisms of docetaxel activity whereas ERK1/2-c-Myc-CXCR4 signaling is implicated in the development of resistance and induction of migration. The aim of this study was to evaluate the hypothesis that the combination treatment with docetaxel and GLIPR1-ΔTM will synergistically induce greater cell death and inhibit the emergence of resistance and development of metastatic potential in prostate cancer (PCa) cells.

**Methods:**

The synergistic effects of the docetaxel and GLIPR1-ΔTM were evaluated with DNA fragmentation, DAPI staining and MTS using paired *t*-test and isobologram study. The effects of the drugs on JNK and ERK1/2-c-Myc-CXCR4 signaling were evaluated with Western blot, DNA fragmentation, and MTS assays using the JNK inhibitor SP600125, and CXCR4 siRNA. The results of docetaxel and GLIPR1-ΔTM combination on migration were examined with scratch assay using the CXCR4 inhibitor AMD3100 while our hypothesis was examined in vivo using VCaP orthotopic xenograft model.

**Results:**

We found that GLIPR1-ΔΤΜ synergized with docetaxel to induce apoptosis in VCaP and PC-3 PCa cells through induction of JNK signaling and concomitant inhibition of ERK1/2-c-Myc-CXCR4 signaling. We showed that JNK activation mediates the apoptotic effects of the drug combination and that CXCR4 knockdown increases its efficacy. We also found that the addition of GLIPR1-ΔΤΜ to docetaxel decreases the migration of VCaP and PC-3 cells. The combination treatment with docetaxel and GLIPR1-ΔTM inhibited tumor growth and decreased metastatic potential in VCaP xenografts more than single agents did.

**Conclusions:**

Our data suggested that addition of GLIPR1-ΔTM treatment in PCa cells increases the efficacy of docetaxel and may inhibit the emergence of drug resistance; potentially permitting a decrease of docetaxel dose for patients with mCRPC eliminating its systemic toxicities.

**Electronic supplementary material:**

The online version of this article (doi:10.1186/s12943-015-0395-0) contains supplementary material, which is available to authorized users.

## Background

The survival of patients with metastatic castration-resistant prostate cancer (mCRPC) remains poor despite the introduction of novel antiandrogens such as enzalutamide and abiraterone [1, 2]. Taxanes, in particular docetaxel and cabazitaxel, are the only chemotherapy agents that have been shown to increase survival in mCRPC patients [3, 4] and recent evidence suggests that these agents are particularly effective in patients with high Gleason score primary disease [5].

Despite the long period of docetaxel use, the exact mechanism(s) of its function is not well-understood. Docetaxel is believed to stabilize microtubules by arresting their de-polymerization, leading to disruption of normal mitosis, G2/M arrest, and inhibition of cell proliferation [6]. This agent inhibits Bcl-2 and Bcl-xL activity through decreased gene expression and posttranslational phosphorylation, promoting apoptosis in PCa cells [6–8]. In melanoma cells, docetaxel induces apoptosis through activation of the c-Jun NH2-terminal kinase (JNK) pathway, but it also activates the ERK1/2 (Extracellular signal-regulated kinase 1/2) signaling which seems to inhibit its apoptotic effects [9]. In addition, docetaxel increases reactive oxygen species (ROS) production, which promotes JNK activation in androgen receptor (AR)- negative PCa cells [10]. Collectively, these data suggest that docetaxel promotes cancer cell death through apoptosis mediated by JNK activation.

Even when mCRPC initially responds to treatment with docetaxel, the disease eventually becomes resistant, which can be attributed to numerous molecular mechanisms according to recent studies in different types of cancer. Mhaidat et al. demonstrated that in melanoma cells treated with docetaxel, activation of PKCδ is associated with proapoptotic responses through JNK activation, as mentioned above, while activation of PKCε enhances pro-survival signaling through ERK1/2 activation [11]. These results indicate that under docetaxel treatment, one pathway downstream of PKC may lead to apoptosis and another associated with ERK signaling may lead to cell survival and resistance to docetaxel. Of note, multiple recent reports demonstrated opposed effects and negative crosstalk between JNK and ERK1/2 signaling [12–15], suggesting that ERK1/2 activation by docetaxel may inhibit its JNK-mediated apoptotic effects. Leonetti et al. found that treatment combining docetaxel with antisense oligodeoxynucleotides against Bcl-2 and c-Myc led to lower PC-3 cell survival in vitro, and had higher antitumor efficacy in PC-3 xenografts, than did docetaxel alone, suggesting that Bcl-2 and c-Myc upregulation may promote resistance to docetaxel as well [16]. Finally, a recent report showed that docetaxel induces the activation of ERK, which stabilizes c-Myc protein, and stimulates CXCR4 (C-X-C chemokine receptor type 4) signaling [17]. The same report showed that CXCR4 can, in turn, activate ERK1/2; these signaling activities establish a positive feedback resistance loop in response to docetaxel [17]. Overall, these findings support the concept that activation of ERK1/2-c-Myc-CXCR4 pathway can promote the development of resistance to docetaxel and potentially inhibit the JNK apoptotic signaling.

Recently, cabazitaxel was shown to be effective in patients who progressed under abiraterone and enzalutamide after failure of docetaxel suggesting that taxanes may still be a reasonable therapeutic approach for these patients [18]. The combination of docetaxel with a novel agent may be the most reasonable approach to increase the apoptotic effects of docetaxel and delay or inhibit the development of resistance. Such combination therapy also has the potential to reduce effective doses of docetaxel, thereby decreasing the incidence of docetaxel side effects such as myelosuppression [19]. Numerous studies failed to show improvement of docetaxel efficacy when it was combined with other targeted agents such as dasatinib and zibotentan [20, 21].

The gene encoding the human glioma pathogenesis-related protein 1 (GLIPR1), a p53 target, is downregulated in PCa due to methylation of its promoter [22, 23]. Li et al. demonstrated that GLIPR1 upregulation leads to accumulation of ROS and subsequent activation of the JNK pathway and downregulation of Bcl-2 [24]. GLIPR1 was also found to be associated with destablizing phosphorylation of β-catenin and c-Myc, leading to their degradation [25]. Our group recently showed that GLIPR1-ΔTM is selectively taken up by PCa cells; activates apoptosis through ROS accumulation; and downregulates c-Myc [26]. Collectively, these results indicate that GLIPR1-ΔTM may be a good candidate for combination therapy with docetaxel, since it promotes PCa specific cell death through JNK activation, whereas it downregulates c-Myc signaling which has been extensively associated with the emergence of resistance to docetaxel through ERK1/2-c-Myc-CXCR4 signaling.

In the current study, we report that GLIPR1-ΔΤΜ increases the sensitivity of PCa cells to docetaxel in a synergistic way through additive induction of JNK-mediated apoptosis in VCaP and PC-3 cells and concurrent inhibition of ERK1/2-c-Myc-CXCR4-mediated development of resistance. The addition of GLIPR1-ΔTM to docetaxel was found to decrease the migration of PCa cells while this combination additively decreased tumor growth and metastatic potential in VCaP xenografts.

## Results

### GLIPR1-ΔΤΜ and docetaxel synergistically decreased survival of VCaP and PC-3 cells in vitro

**Fig. 1:**
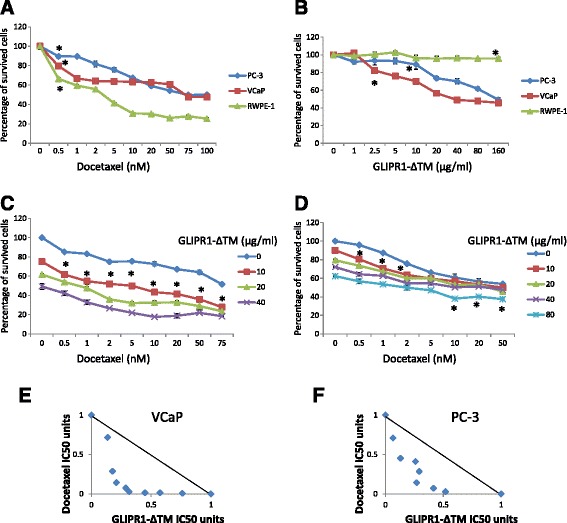
GLIPR1-ΔTM reduced VCaP and PC-3 cells’ survival synergistically with docetaxel. **a**. Docetaxel dose–response curves of VCaP, PC-3, and RWPE-1 cells treated for 48 h, determined using MTS assay. At 0.5nM, docetaxel significantly decreased cell survival in the three cell lines. The IC50 was 69.8nM for VCaP cells and 70.5nM for PC-3 cells. **b**. GLIPR1-ΔTM dose–response curves of VCaP, PC-3, and RWPE-1 cells treated for 48 h, determined using MTS assay. At 2.5 μg/ml and 10 μg/ml, GLIPR1-ΔTM significantly decreased survival of VCaP and PC-3 cells, respectively. Only 160 μg/ml decreased survival of RWPE-1 cells. The IC50 was 34.8 μg/ml for VCaP cells and 154 μg/ml for PC-3 cells. **c**. The addition of 10 μg/ml GLIPR1-ΔTM increased the efficacies of all doses of docetaxel in inhibiting VCaP cells’ survival, based on MTS assay. **d**. The addition of 10 μg/ml GLIPR1-ΔTM increased the efficacies of 0.5, 1, and 2nM docetaxel in inhibiting PC-3 cells’ survival as determined by MTS assay, and the addition of 80 μg/ml GLIPR1-ΔTM increased the efficacies of 10, 20, and 50nM docetaxel. **e**. Isobologram analysis showed that GLIPR1-ΔTM and docetaxel synergistically induced cell death in VCaP cells. **f**. Isobologram analysis showed that GLIPR1-ΔTM and docetaxel induced cell death synergistically in PC-3 cells. The results are presented as the mean ± standard error from at least three independent experiments

To test the hypothesis that GLIPR1-ΔTM sensitizes PCa cells to docetaxel, we used VCaP cells and PC-3 cells. Both cell lines were derived from bone metastases of PCa. VCaP cells were derived from a patient with mCRPC, while PC-3 cells are androgen receptor-negative metastatic PCa cells. We also included RWPE-1 cells, which are epithelial cells derived from the peripheral zone of an histologically normal adult human prostate and transfected with a single copy of human papillomavirus 18 [26]. The three cell lines were treated for 48 h with various concentrations of docetaxel (0.5, 1, 2, 5, 10, 20, 50, 75, and 100nM) and GLIPR1-ΔΤΜ (1, 2, 5, 10, 20, 40, 80,and 160 μg/ml) in different serum concentrations (0.1 % for PC-3 cells and 0.5 % for VCaP and RWPE-1 cells), and MTS assay was performed to evaluate the dose response and the IC50 for each single-agent treatment. At a concentration of 0.5nM, docetaxel resulted in significantly lower survival of both PCa cell lines (*p* = 0.004 for VCaP cells, *p* = 0.03 for PC-3 cells) than did the control treatment (Fig. 1a). The IC50 was 69.8nM for VCaP cells and 70.5nM for PC-3 cells. Docetaxel significantly decreased survival of normal prostate cells (RWPE-1) even at the lowest (0.5nM) concentration (*p* < 0.0001) (Fig. 1a). At 2.5 μg/ml GLIPR1-ΔΤΜ resulted in significantly decreased survival of VCaP cells (*p* = 0.002), and at 10 μg/ml resulted in significantly decreased survival of PC-3 cells (*p* = 0.0005) compared to control treatment (Fig. 1b). The IC50 was 34.8 μg/ml for VCaP cells and 154 μg/ml for PC-3 cells. Interestingly, only 160 μg/ml GLIPR1-ΔTM decreased survival of normal prostate cells (RWPE-1) (*p* = 0.002) (Fig. 1b), which is consistent with our previous results [25] showing that GLIPR1-ΔTM is selectively taken up by cancerous cells, promoting their apoptosis, while normal prostate cells are not sensitive to this agent.

To investigate the potential synergy of docetaxel and GLIPR1-ΔTM in inducing anti-cancer effects, we treated VCaP and PC-3 cells with various combinations of the two agents: 0.5, 1, 2, 5, 10, 20, 50, and 75nM docetaxel in both cell lines; 0, 10, 20, and 40 μg/ml GLIPR1-ΔTM in VCaP cells; and 0, 10, 20, 40, and 80 μg/ml GLIPR1-ΔTM in PC-3 cells. At 10 μg/ml, GLIPR1-ΔTM significantly increased the efficacy of docetaxel in VCaP cells at all docetaxel doses (Fig. 1c) and significantly increased the efficacy of docetaxel at 0.5, 1, and 2nM docetaxel in PC-3 cells (Fig. 1d). At 80 μg/ml, GLIPR1-ΔTM significantly increased the efficacy of higher doses of docetaxel (10, 20, and 50nM) (Fig. 1d).

To evaluate whether the effects of combination treatments on survival were super-additive (synergistic), 50 % of maximum efficacy was used to determine the fixed ratio, and multiple combination doses with the same efficacy were included. Isobologram analysis showed that the effects were synergistic (Fig. 1e, f).

### GLIPR1-ΔTM increased the apoptotic effect of docetaxel in VCaP and PC-3 cells

**Fig. 2:**
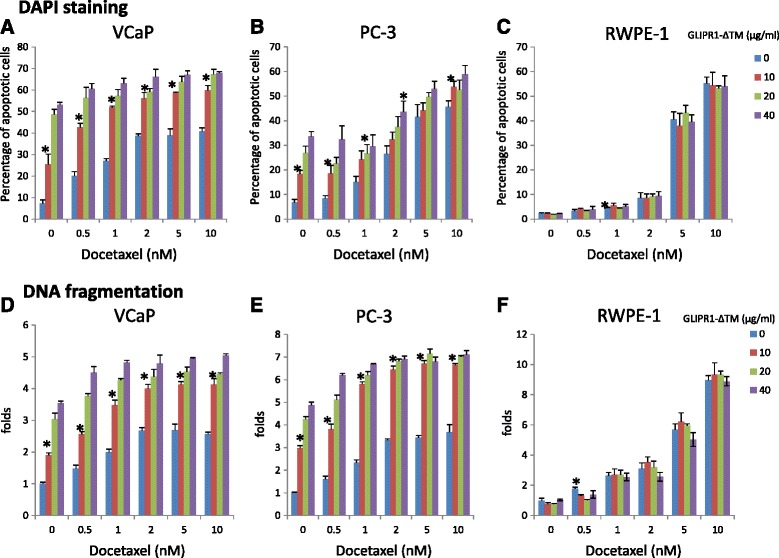
GLIPR1-ΔTM increased the apoptotic effect of docetaxel in VCaP and PC-3 cells. **a**. The addition of 10 μg/ml GLIPR1-ΔΤΜ increased the apoptotic effects of all docetaxel’s doses in VCaP cells, measured by DAPI staining. **b**. The addition of 10 μg/ml GLIPR1-ΔΤΜ increased the apoptotic effects of all docetaxel doses in PC-3 cells, measured by DAPI staining. **c**. GLIPR1-ΔΤΜ did not increase the apoptotic effect of any docetaxel dose in RWPE-1 cells, while 1nM docetaxel significantly increased the percentage of apoptotic cells, based on DAPI staining. **d**. The addition of 10 μg/ml GLIPR1-ΔΤΜ increased the apoptotic effects of all docetaxel doses in VCaP cells, measured by DNA fragmentation. **e**. The addition of 10 μg/ml GLIPR1-ΔΤΜ increased the apoptotic effects of all docetaxel doses in PC-3 cells, measured by DNA fragmentation. **f**. GLIPR1-ΔΤΜ did not increase the apoptotic effect of any docetaxel dose in RWPE-1 cells, while 0.5nM docetaxel significantly increased the percentage of apoptotic cells, based on DNA fragmentation. The results are presented as the mean ± standard error from at least three independent experiments

Given the known apoptotic effects of GLIPR1-ΔTM and docetaxel in PCa cells, we hypothesized that the decreased survival of PCa cells treated with combinations of these two agents is mainly due to increased apoptosis. To test this hypothesis, we used DAPI staining and DNA fragmentation assay to examine apoptosis in VCaP and PC-3 cells. We included RWPE-1 cells as a control. VCaP and RWPE-1 cells were treated with 0.5 % serum-containing medium for 24 h, and PC-3 cells were treated with 0.1 % serum-containing medium for 24 h. Then, cells were treated with various concentrations of GLIPR1-ΔTM (0, 10, 20, and 40 μg/ml) for 1 h followed by addition of various concentrations of docetaxel (0, 0.5, 1, 2, 5, and 10nM). Forty-eight hours later, DAPI staining was evaluated. At 10 μg/ml, GLIPR1-ΔTM in combination with all docetaxel concentrations significantly increased the percentage of apoptotic VCaP cells (*p* < 0.001) (Fig. 2a). In PC-3 cells, 10 μg/ml GLIPR1-ΔTM significantly increased the apoptotic effects of 0.5nM (*p* = 0.014) and 10nM (*p* = 0.033) docetaxel, while 20 μg/ml GLIPR1-ΔTM significantly increased the apoptosis induced by 1nM docetaxel (*p* = 0.016) and 40 μg/ml GLIPR1-ΔΤΜ increased the apoptotic effect of 2nM docetaxel (*p* = 0.0095) (Fig. 2b). In RWPE-1 cells, GLIPR1-ΔTM did not increase the apoptosis induced by any dose of docetaxel, which is consistent with the results of the cell survival assay and our previous data showing that GLIPR1-ΔTM selectively induces apoptosis in PCa cells but not in normal prostate cells. In RWPE-1 cells, 1nM docetaxel induced apoptosis (*p* = 0.0025) (Fig. 2c).

According to DNA fragmentation assay, 10 μg/ml GLIPR1-ΔTM in combination with all docetaxel doses increased apoptosis in VCaP cells (*p* = 0.0014 for 0.5, 1, and 2nM docetaxel; *p* = 0.0028 for 5nM docetaxel; *p* = 0.0012 for 10nM docetaxel) (Fig. 2d). In PC-3 cells, 10 μg/ml GLIPR1-ΔTM enhanced the apoptotic effects of all doses of docetaxel (*p* = 0.001 for 0.5nM docetaxel; *p* < 0.001 for 1, 2, 5, and 10nM docetaxel) (Fig. 2e). GLIPR1-ΔTM did not increase the apoptotic effects of any docetaxel doses in RWPE-1 cells, in which 0.5nM docetaxel induced apoptosis (*p* = 0.0094) (Fig. 2f). Collectively, these results demonstrated that GLIPR1-ΔTM enhanced the apoptotic activity of docetaxel in VCaP and PC-3 PCa cells without substantially affecting normal prostate cells.

### Combination treatment with docetaxel and GLIPR1-ΔTM induced JNK signaling and inhibited the docetaxel-induced ERK1/2-c-Myc-CXCR4 signaling resistance loop, increasing apoptosis through maximal JNK activation

**Fig. 3:**
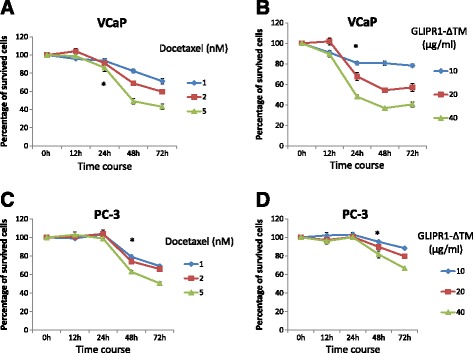
Time-dependent curves of docetaxel and GLIPR1-ΔTM efficacies in VCaP and PC-3 cells treated with 1, 2, or 5nM docetaxel or 10, 20, or 40 μg/ml GLIPR1-ΔTM. **a**,**b**. 1nM docetaxel and 10 μg/ml GLIPR1-ΔΤΜ significantly decreased survival of VCaP cells at 24 h (*p* = 0.03 for docetaxel and *p* = 0.001 for GLIPR1-ΔTM). **c**,**d**. Time-dependent curves of docetaxel and GLIPR1-ΔTM efficacies in PC-3 cells treated with 1, 2, or 5nM docetaxel or 10, 20, or 40 μg/ml GLIPR1-ΔTM showed that 1nM docetaxel and 10 μg/ml GLIPR1-ΔΤΜ significantly decreased survival of these cells at 48 h (*p* < 0.001 for docetaxel and *p* = 0.007 for GLIPR1-ΔTM). The results are presented as the mean ± standard error from at least three independent experiments

We have previously demonstrated that endogenous GLIPR1 activates JNK signaling [22] and docetaxel was also found to induce JNK signaling and subsequent apoptosis in melanoma cells [9]. Initially, we hypothesized that the greater apoptotic effect of the combination of these two agents is mediated by JNK activation and inhibition of ERK1/2-c-Myc-CXCR4 signaling by GLIPR1-ΔTM. To test this hypothesis, we initially examined the effect of combination treatment on JNK and ERK1/2-c-Myc-CXCR4 signaling. To select the right time-point for this evaluation during treatment using the lowest effective doses, we examined survival of VCaP and PC-3 cells treated with 1, 2, or 5nM docetaxel or 10, 20, or 40 μg/ml GLIPR1-ΔTM. The time-response curves were different in VCaP and PC-3 cells. In particular, 1nM docetaxel and 10 μg/ml GLIPR1-ΔTM significantly decreased the survival of VCaP cells at 24 h (*p* = 0.03 for docetaxel and *p* = 0.001 for GLIPR1-ΔTM) (Fig. 3a) and the survival of PC-3 cells at 48 h (*p* < 0.001 for docetaxel and *p* = 0.007 for GLIPR1-ΔTM) (Fig. 3b).

**Fig. 4:**
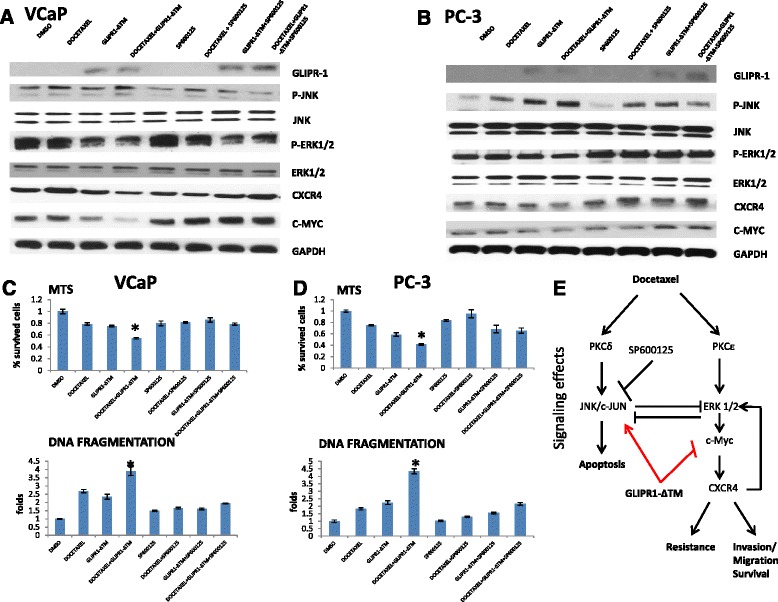
GLIPR1-ΔΤΜ synergized with docetaxel in activating JNK/c-Jun through JNK phosphorylation, whereas addition of GLIPR1-ΔΤΜ to docetaxel reduced ERK1/2 signaling. **a**,**b**. VCaP and PC-3 cells were treated with 1nM docetaxel, 10 μg/ml GLIPR1-ΔTM, or both and then, we added JNK inhibitor (SP600125) 1 μΜ to single agents or to their combination. Total treatment administration lasted for 24 h (VCaP cells) or 48 h (PC-3 cells), and the effects on JNK and ERK1/2 signaling were evaluated via western blot. JNK phosphorylation was increased synergistically with the combination of docetaxel and GLIPR1-ΔΤΜ, a pathway that leads to apoptosis; whereas ERK1/2 phosphorylation, which results in drug resistance and migration through c-Myc-CXCR4, was reduced by administration of this combination. These activities were reversed by the JNK inhibitor, SP600125. Western blot experiments were conducted three times and the quantitative data are presented as supplementary data. **c**,**d**. Under the same conditions and treatments in both cell lines, we performed MTS and DNA fragmentation assay to evaluate the percentage of survived cells and cell apoptosis, respectively. We found that, in both cell lines, the combination treatment of docetaxel and GLIPR1-ΔΤΜ resulted in statistically significant decrease in the percentage of survived cells and increase of apoptotic cells (VCaP: *p* < 0.001 compared to both single agents in MTS assay, *p* = 0.02 compared to GLIPR1-ΔΤΜ and *p* < 0.001 compared to docetaxel according to DNA fragmentation assay, PC-3: *p* = 0.001 compared to both single agents in MTS assay, *p* < 0.001 compared to both single agents according to DNA fragmentation assay) but this observation was reversed significantly when we added the JNK inhibitor (SP600125) to the combination docetaxel and GLIPR1-ΔΤΜ (*p* < 0.001 for both cells lines according to both techniques). The results are presented as the mean ± standard error from at least three independent experiments. **e**. Signaling effects of the combination treatment of docetaxel and GLIPR1-ΔΤΜ. Docetaxel induces JNK phosphorylation (apoptosis pathway); whereas it concomitantly induces ERK1/2 phoshorylation (drug resistance and migration pathway). JNK and ERK1/2 pathways can demonstrate reciprocal inhibition. GLIPR-ΔΤΜ induces JNK signaling but inhibits the ERK1/2-c-Myc-CXCR4 resistance loop. Thus, docetaxel and GLIPR1-ΔΤΜ combination treatment leads to JNK pathway dominance over the ERK1/2 pathway, and apoptosis dominates over drug resistance and invasion/migration

We next investigated the effect of the combination of 1nM docetaxel and 10 μg/ml GLIPR1-ΔTM on JNK and ERK1/2-c-Myc-CXCR4 signaling at 24 h for VCaP cells and 48 h for PC-3 cells. In order to evaluate the role of JNK signaling in apoptosis and ERK1/2-c-Myc-CXCR4 loop, we included the JNK inhibitor SP600125 in these experiments, a known agent which acts through inhibiting JNK phosphorylation [27]. VCaP and PC-3 cells were treated with 0.5 % or 0.1 % serum-containing medium, respectively, for 24 h. Then, cells were treated with 10 μg/ml GLIPR1-ΔTM for 1 h followed by addition of 1nM docetaxel with or without 1 μM SP600125 for 24 or 48 h. Western blot experiments were conducted at least three times and our results reflect at least three independent experiments. The quantitative data are presented as supplementary data (Additional file 1: Figure S1). Representative blots are presented in the Fig. 4. Combination treatment increased JNK phosphorylation in VCaP and PC-3 cells more than docetaxel or GLIPR1-ΔTM did when used as single agents (Fig. 4a, b). Based on our Western blot data, docetaxel alone increased the ERK1/2 phosphorylation (modestly in VCaP cells), and c-Myc and CXCR4 protein levels whereas the combination treatment reduced ERK1/2 phosphorylation and c-Myc and CXCR4 protein levels in both cell lines. In contrast, the addition of SP600125 to the combination treatment increased ERK1/2 phosphorylation and protein levels of c-Myc and CXCR4 (Fig. 4a, b).

To further test our hypothesis that JNK mediates the apoptotic effects of docetaxel and GLIPR1-ΔΤΜ combination treatment, we treated VCaP and PC-3 cells with DMSO, docetaxel, GLIPR1-ΔΤΜ or combination treatment with or without JNK inhibitor, SP600125, and performed MTS and DNA fragmentation assays to evaluate survival and apoptosis, respectively. We found that the percentage of survived VCaP and PC-3 cells treated with the combination of docetaxel and GLIPR1-ΔTM was significantly increased when SP600125 was added (*p* < 0.001 for both cell lines) according to MTS assay. Additionally, in PC-3 cells, we also found that the addition of SP600125 increased the survival when added to docetaxel (*p* = 0.048) which is consistent with the JNK-mediated apoptotic effect of docetaxel. Through DNA fragmentation assay, we found that the addition of SP600125 decreased the apoptotic effect of docetaxel (*p* < 0.001 for VCaP and PC-3 cells), GLIPR1-ΔTM (*p* < 0.001 for VCaP cells and *p* = 0.002 for PC-3 cells) and combination therapy (*p* < 0.001 for VCaP and PC-3 cells). Collectively, these data suggested that docetaxel and GLIPR1-ΔΤΜ combination treatment synergistically increased apoptosis by maximal activation of JNK, together with suppression of ERK1/2 signaling, which further increased JNK signaling through a derepression mechanism. Finally, the ERK1/2-c-Myc-CXCR4 docetaxel-induced resistance loop [17], was downregulated by the drug combination which led to reduced migration activity.

### CXCR4 knockdown potentiated the apoptotic effects of docetaxel and the combination treatment with docetaxel and GLIPR1-ΔTM decreased the migration of VCaP and PC-3 cells

**Fig. 5:**
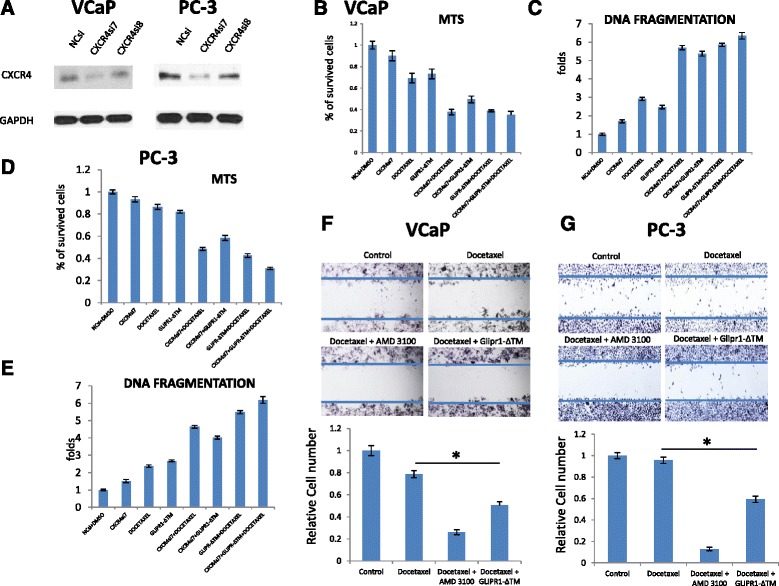
CXCR4si increased the sensitivity of VCaP and PC-3 cells to docetaxel and reduced cell migration trend when combined with docetaxel. **a**. Western blot in VCaP and PC-3 cells to evaluate the efficacy of CXCR4si7 and CXCR4si8 siRNAs. The results are presented as the mean ± standard error from three independent experiments. Quantitative data of western blots on 2 different CXCR4siRNAs for validation and determination of their effect size are provided in Supplementary Data. **b**. CXCR4 knockdown decreased the survival of VCaP cells when was combined with GLIPR1-ΔTM (*p* < 0.001), docetaxel (*p* < 0.001) and combination (*p* = 0.004) than the CXCR4 knockdown alone did according to MTS assay. **c**. CXCR4 knockdown increased the apoptosis of VCaP when was combined with GLIPR1-ΔTM (*p* < 0.001), docetaxel (*p* < 0.001) and combination (*p* = 0.041) than the CXCR4 knockdown did according to DNA fragmentation assay. The results are presented as the mean ± standard error from at least three independent experiments. **d**. CXCR4 knockdown decreased the survival of PC-3 cells when was combined with GLIPR1-ΔTM (*p* < 0.001), docetaxel (*p* < 0.001) and combination (*p* = 0.01) than the CXCR4 knockdown alone did according to MTS assay. **e**. CXCR4 knockdown increased the apoptosis of PC-3 cells when combined with GLIPR1-ΔTM (*p* < 0.001), docetaxel (*p* < 0.001) and combination (*p* < 0.001) than the CXCR4 knockdone alone did. The results are presented as the mean ± standard error from at least three independent experiments. **f**. Migration of VCaP cells was evaluated by scratch assay after 24 h of treatment with 1nM docetaxel with or without the addition of 10 μg/ml GLIPR1-ΔΤΜ or 25 μg/ml AMD3100, a known CXCR4 inhibitor. The addition of GLIPR1-ΔTM significantly decreased the number of migrated cells (*p* < 0.001). **g**. Migration of PC-3 cells was evaluated by scratch assay after 24 h of treatment with 1nM docetaxel with or without the addition of 10 μg/ml GLIPR1-ΔΤΜ or 25 μg/ml AMD3100. The addition of GLIPR1-ΔTM significantly decreased the number of migrated cells (*p* < 0.001). The migration assay experiments were conducted twice and the presented results are the averages of these two independent experiments

We showed that the addition of GLIPR1-ΔTM decreased the docetaxel-mediated ERK1/2-c-Myc-CXCR4 induction in VCaP and PC-3 cells. GLIPR1-ΔΤΜ was expected to further activate the apoptosis and inhibited the development of resistance through this pathway. We evaluated the effects of the CXCR4 downregulation on docetaxel and GLIPR1-ΔTM treatment in terms of apoptosis and survival of VCaP and PC-3 cells. Thus, we treated VCaP and PC-3 cells with CXCR4si7 and CXCR4si8 siRNAs for 48 h and found that the most effective siRNA in terms of downregulation of CXCR4 is the CXCR4si7. Consequently, we treated VCaP and PC-3 cells with CXCR4si7 for 24 h and then treated them with GLIPR1-ΔΤΜ and docetaxel for 24 and 48 h as described above. Western blot experiments were conducted three times and the quantitative data are presented as supplementary data (Additional file 1: Figure S1). We evaluated the survival with MTS assay and apoptosis with DNA fragmentation assay. We found that the treatment with CXCR4si reduced the survival of cells treated with docetaxel (*p* < 0.001), GLIPR1-ΔΤΜ (*p* < 0.001) and combination (*p* = 0.041) (Fig. 5b) in VCaP cells. According to DNA fragmentation assay, we found that knockdown of CXCR4 increased the apoptotic effect of docetaxel (*p* < 0.001), GLIPR1-ΔΤΜ (*p* < 0.001) and combination (*p* = 0.01) in VCaP cells (Fig. 5c). In PC-3 cells, we found that CXCR4 downregulation reduced the survival of cells treated with docetaxel (*p* < 0.001), GLIPR1-ΔΤΜ (*p* < 0.001) and combination (*p* < 0.001) (Fig. 5d). According to DNA fragmentation assay, we found that CXCR4 knockdown increased the apoptotic effect of docetaxel (*p* < 0.001), GLIPR1-ΔΤΜ (*p* < 0.001) and combination (*p* < 0.001) in PC-3 cells (Fig. 5e). These data suggested that CXCR4 downregulation further potentiated the apoptotic effect of each single agent and their combination as well, when was combined with them, confirming that the inhibition of the ERK1/2-c-Myc-CXCR4 resistance pathway led to increased apoptosis. In our proposed combination treatment, GLIPR1-ΔΤΜ worked as the key molecule which reduced CXCR4 levels through suppression of ERK1/2-c-Myc, and consequently maximally increased JNK-induced apoptotic signaling stimulated by docetaxel through ERK1/2 downregulation and JNK derepression. Given the known role of CXCR4 as an inducer of migration and metastasis in PCa [28], and based on our results that GLIPR1-ΔTM inhibited the docetaxel-induced ERK1/2-c-Myc-CXCR4 signaling, we evaluated the hypothesis that the combination of GLIPR1-ΔΤΜ and docetaxel can inhibit migration of VCaP and PC-3 cells more than docetaxel alone. We treated VCaP and PC-3 cells with 0.5 % or 0.1 % serum-containing medium, respectively, followed by 1nM docetaxel, with or without the addition of 10 μg/ml GLIPR1-ΔΤΜ hour before the initiation of docetaxel treatment. After 24 h cell migration was assessed by scratch assay. As a positive control, we used 25 μg/ml AMD3100, a CXCR4 inhibitor, as previously described [29]. The addition of GLIPR1-ΔΤΜ to docetaxel significantly decreased the number of migrated VCaP cells (*p* < 0.001) (Fig. 5f) and PC-3 cells (*p* < 0.001) than docetaxel alone did (Fig. 5g).

### Combination treatment with docetaxel and GLIPR-ΔΤΜ inhibited growth and metastasis of VCaP othotopic xenografts

**Fig. 6:**
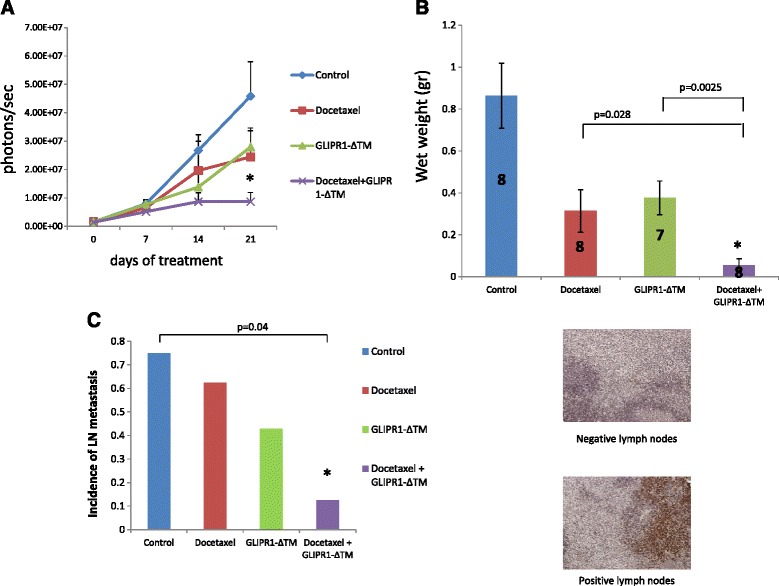
Docetaxel GLIPR1-ΔΤΜ combination treatment inhibited growth and metastasis in VCaP orthotopic xenografts. **a**. Nude mice bearing VCaP xenografts were treated with docetaxel, GLIPR1-ΔΤΜ, or both. Combination treatment significantly decreased the IVIS signal (photons/s) than GLIPR1-ΔΤΜ alone did (*p* = 0.012) but docetaxel alone did not (*p* = 0.16). **b**. Combination treatment decreased significantly the wet weight of VCaP xenografts rather thad GLIPR1-ΔΤΜ alone (*p* = 0.0025) and docetaxel alone (*p* = 0.028) did. **c**. Mouse lymph node tissues were stained for cytokeratin 18 to evaluate the presence of metastatic VCaP cells. The combination of docetaxel and GLIPR1-ΔΤΜ resulted in a lower incidence of LN metastasis than control treatment did (*p* = 0.04), whereas single-agent treatments did not result in a significant reduction

To validate the efficacy of the combination of GLIPR1-ΔTM and docetaxel in vivo, we treated nude mice bearing VCaP orthotopic tumors with PBS (control), docetaxel alone, GLIPR1-ΔTM alone, or the combination of these two agents. Three weeks after the initiation of treatment, combination treatment significantly decreased the IVIS signal (photons/second) than GLIPR1-ΔTM alone did (*p* = 0.012) but docetaxel alone did not (*p* = 0.16) (Fig. 6a). However, the tumors that were treated with combination therapy had significantly lower wet weight than the tumors in the mice treated with docetaxel alone (*p* = 0.028) or GLIPR1-ΔTM alone (*p* = 0.0025) did (Fig. 6b). We collected lymph node samples from all mice and evaluated them for the presence of metastatic PCa cells as evidenced by cytokeratin 18 staining. Neither of the two agents significantly altered the emergence of metastasis, but combination therapy decreased the incidence of metastasis than control treatment did (*p* = 0.04) (Fig. 6c).

## Discussion

In the present study we tested our hypothesis that GLIPR1-ΔΤΜ can synergize with docetaxel (one of the two chemotherapy agents approved for the treatment of mCRPC) and whether the combination treatment can lead to greater cell death than treatment with docetaxel alone does. We showed that the combination of these two agents had synergistic effects on decreasing cell survival of VCaP and PC-3 cells. Apoptosis was the main mechanism for cell death, as demonstrated with DAPI staining and DNA fragmentation assay. Of note, the combination treatment appeared to lead to additive induction of JNK signaling since GLIPR1-ΔΤΜ was found to inhibit the ERK1/2-c-Myc-CXCR4 resistance loop and consequently to suppress the inhibitory effects of ERK1/2 on JNK signaling, driving JNK signaling. Thus, the efficacy of the combination of these two agents is suggested to be mediated through synergistic apoptotic activity driven by dominant JNK signaling and downregulation of ERK1/2-c-Myc-CXCR4-mediated drug resistance and migration.

We further tested our hypothesis regarding the JNK hyperactivation via JNK inhibitor administration using two biological assays to assess the effect on survival and apoptosis and western blot to monitor the signaling changes. We found that the addition of the inhibitor to the combination treatment resulted in increased survival and reduced apoptosis compared to combination treatment alone in both PCa cell lines; whereas on Western blots we observed a marked reduction in JNK phosphorylation together with a concomitant increase in ERK1/2 phosphorylation, and c-Myc and CXCR4 protein levels compared to combination treatment without inhibitor. We further tested the effect of CXCR4 downregulation on combination treatment to determine its role as a central component of the ERK1/2-c-Myc-stimulated resistance loop. In support of our hypothesis, we found that CXCR4 knockdown combined with the combination treatment further enhanced apoptosis and reduced the survival of the cells. This finding demonstrates the opposing biological effects of GLIPR1-ΔΤΜ on c-Myc-CXCR4-mediated drug resistance and migration [17, 28]. In consideration of this mechanism we tested our hypothesis via migration assay and found that docetaxel and GLIPR1-ΔΤΜ combination treatment resulted in significantly reduced numbers of migrated cells than docetaxel alone did, mimicking the result of docetaxel combined with CXCR4 inhibitor and supporting our hypothesis for a role for GLIPR1-ΔΤΜ suppression of PCa cell migration.

To test our treatment combination effectiveness in vivo, we treated nude mice bearing VCaP orthotopic xenografts with docetaxel, GLIPR1-ΔΤΜ, or both for 3 weeks. Combination treatment decreased IVIS signal than GLIPR1-ΔΤΜ did and decreased significantly tumor weight than both single-agent treatments did. The finding that the incidence of lymph node metastasis was significantly lower in animals treated with the combination treatment than in control-treated animals is consistent with our in vitro data and may be related to the inhibition of the docetaxel-induced ERK1/2-CXCR4-c-Myc axis by GLIPR1-ΔTM.

## Conclusions

The results of our study demonstrated that docetaxel and GLIPR1-ΔΤΜ combination treatment leads to increased JNK-mediated apoptosis compared to single agent treatment. These activities are accompanied by inhibition of ERK1/2-c-Myc-CXCR4 signaling, which results in derepression of JNK signaling, leading to maximal JNK-mediated apoptotic effects. In addition docetaxel and GLIPR1-ΔΤΜ combination treatment may suppress migration through downregulation of the ERK1/2-c-Myc-CXCR4 drug resistance pathway and reduces metastatic potential in vivo. Based on our results, its conceivable that GLIPR1-ΔΤΜ (which is currently progressing toward clinical trials) or therapy agents that act through similar mechanisms may be used together with reduced dosage of docetaxel with potentially greater therapeutic efficacy than docetaxel alone, and with probably fewer adverse effects. It is also conceivable that docetaxel and GLIPR1-ΔΤΜ combination treatment could possibly result in delay in developing docetaxel resistance. Further preclinical and clinical studies will be needed to develop GLIPR1-ΔΤΜ therapy through clinical trials and to evaluate the safety and efficacy of this combination in patients with mCRPC.

## Methods

### Cell lines and reagents

The non-tumorigenic RWPE-1 cells (from the American Type Culture Collection) were grown in complete keratinocyte serum-free medium. PC-3 and VCaP prostate cancer cells were grown as described previously [26]. All cell lines were validated by short tandem repeat DNA fingerprinting with the AmpFlSTR Identifier Kit (Applied Biosystems, Inc., Foster City, CA) at the Characterized Cell Line Core Facility of The University of Texas MD Anderson Cancer Center. Docetaxel and AMD3100, a CXCR4 inhibitor, were purchased from Sigma-Aldrich (St. Louis, MO). SP600125, a JNK inhibitor, was purchased from Santa Cruz.

### Purification of recombinant GLIPR1-ΔΤΜ

GLIPR1-ΔTM coding sequence was obtained from normal prostate tissue by RT-PCR using specific primers (forward: 5′CCCAAGCTTGCAAATATTTTGCCAGAT3′, reverse: 5ATAGT TTAGCGGCCGCTCTGTTACGTGGATATAT3′). The PCR product was digested with restriction enzymes Hinlll and NotI and inserted into pSectag/ Hygro2 Hinlll and NotI sites to generate pSec-GLIPR1-ΔTM as previously described [24, 26]. Conditioned medium from 293 Freestyle cells transfected with pSec-GLIPR1-ΔTM was collected and centrifuged, and GLIPR1-ΔTM was purified using Ni-NTA agarose.

### Cell viability assay

Cell viability was analyzed using an MTS CellTiter 96 Aqueous One Solution Cell Proliferation Assay (Promega, Madison, WI) according to the manufacturer’s instructions as previously described [30]. After appropriate cell treatment (96-well assay plate) 20 μl of CellTiter 96® Aqueous One Solution Reagent were added into each well. The plate was intubated for 2 h at 37 °C in a humidified 5 % CO_2_ chamber. The absorbance at 490 nm was recorded using the 96-well plate reader.

### Apoptosis analysis

Apoptotic nuclear morphology was evaluated with fluorescence microscopy after 4′,6-diamidino-2-phenylindole (DAPI) staining (2 μg/ml). DNA fragmentation analysis was performed using Cell Death Detection ELISA (Roche Applied Science, Indianapolis, IN) according to the manufacturer’s instructions. After the appropriate treatment, a cell suspension of 2 × 10^5^ cells was collected, the cells were pelleted by centrifugation and the supernatant was discarded. 1 mL of PBS was added and the full volume of re-suspended cells was transferred to 4 mL of absolute ethanol at −20 °C by pipetting the cell suspension slowly into the ethanol while vortexing at top speed. The cells were intubated in ethanol at −20 °C for 5–15°min. The ethanol was discarded and 5 mL of PBS at room temperature to rehydrate for 15 min. The DAPI stock solution was diluted to 3 μM in staining buffer (100 mM Tris, pH 7.4, 150 mM NaCl, 1 mM CaCl2, 0.5 mM MgCl2, 0.1 % Nonidet P-40). After adding the solution the cell suspension was centrifuged and the supernatant was discarded. 1 mL of DAPI diluted in staining buffer was added and the cells were intubated for 15 min at room temperature. The cells’ staining was analyzed by flow cytometry.

### DNA fragmentation assay

DNA fragmentation assay was performed using a Cell Death Detection ELISA Kit (Roche, Mannheim, Germany) according to manufacturer’s protocol as previously described [26]. The assay is a photometric enzyme-immunoassay for the qualitative and quantitative in vitro determination of cytoplasmic histone-associated DNA fragments (mono- and oligonucleosomes) after induced cell death.

### Isobologram analysis

The effects of drug combinations were categorized as additive, synergistic, or antagonistic by isobologram analysis as previously described [31], and IC50 was used to determine the fixed ratio. A graph of equally effective dose pairs (isoboles) for a single effect level is presented. Specifically, a particular effect level of 50 % of the maximum is selected, and doses of docetaxel and GLIPR1-ΔTM (each alone) that give this effect are plotted as axial points in a Cartesian plot. The straight line connecting axial and vertical axes is the locus of points (dose pairs) that will produce this effect in a simply additive combination. This line of additivity allows a comparison with the actual dose pair that produces this effect level experimentally. An actual dose pair below the straight line attains this effect with lesser quantities and is super-additive (synergistic), while the dose pair denoted above the straight line means greater quantities are required and is therefore sub-additive.

### RNA interference

Knockdown of CXCR4 was achieved by transient transfection of VCaP cells with a pool of CXCR4-specific siRNA (Invitrogen), and a pool of non-targeting siRNA (NCsi) (Invitrogen), as control, by using Lipofectamine RNAiMax transfection reagent (Invitrogen). VCaP cells were seeded at a density of 1.0 × 10^6^ in six-well plates. Cells were transfected with 20nM CXCR4si or NCsi the following day. 24 h later cells were treated with DMSO or 1 and 2nM of docetaxel in Dulbecco modified Eagle medium (DMEM) containing 0.5 % serum.

### Western blot analysis

For evaluation of JNK and ERK1/2-c-Myc-CXCR4 signaling, PC-3 cells were treated for 24 h with RPMI-1640 medium containing 0.1 % serum and VCaP cells were treated for 24 h with Dulbecco modified Eagle medium (DMEM) containing 0.5 % serum. Then, cells were treated with 10 μg/ml GLIPR1-ΔΤΜ for 1 h followed by the addition of 1nM docetaxel with or without 1μΜ SP600125 and lysates were collected 24 h later (VCaP cells) and 48 h later (PC-3 cells). Antibodies against GLIPR1-ΔΤΜ (Myc-tag), phospho-JNK, JNK, phospho-ERK1/2, ERK1/2, c-Myc, and CXCR4 were all purchased from Cell Signaling Technology (Danvers, MA), and antibody against GAPDH was purchased from Santa Cruz. When indicated, densitometric analysis was performed and quantification of integrated density was assessed using the NIS-Elements-AR software program (version 3.0; Nikon) followed by GAPDH normalization.

### Scratch assay

PC-3 cells (1 × 10^5^) and VCaP cells (2 × 10^5^) were seeded in six-well plates and incubated overnight to achieve confluency. Next day, PC-3 cells were treated for 24 h with RPMI-1640 medium containing 0.1 % serum, and VCaP cells were treated for 24 h with DMEM containing 0.5 % serum. Then, the cell layer in each well was scratched using a plastic pipette tip, and cells were treated with 10 μg/ml GLIPR1-ΔΤΜ for 1 h followed by the addition of 1nM docetaxel for 24 h. Cells were visualized under a microscope and photographed for the evaluation of migration.

### Tumor induction in mice, treatment protocols, and immunohistochemical analysis

VCaP cells were transduced with lentivirus stably expressing luciferase. Aliquots of 2 × 10^6^ VCaP-luciferase cells in 25 μl of PBS were injected directly into the right lobe of the dorsolateral prostate in athymic nude male mice (Taconic Farms, Hudson, NY) to induce orthotopic tumors. The tumors were allowed to grow for 14 days before treatment. Mice were treated with 10 mg/kg docetaxel intraperitonealy weekly, 20 μg of GLIPR1-ΔTM intraperitonealy three times per week, or both. The control group was treated with PBS. Tumor size was monitored by measuring the luminescence signal using the IVIS 200 imaging system (PerkinElmer, Wellesley, MA). After 3 weeks of treatment, tumor-bearing mice were sacrificed, and the tumors were collected and weighed. Lymph nodes were also collected, and antibody to cytokeratin 18 (DAKO, Carpinteria, CA; catalog no. M701029-2) was used for immunostaining on formalin-fixed paraffin-embedded lymph node tissues. All tissue sections were processed by using an avidin–biotin peroxidase complex kit (Vector Laboratories, Burlingame, CA) as previously described [32].

### Statistical analysis

The results are presented as the mean ± standard error from at least three independent experiments. Comparisons of groups were appropriately analyzed using the Student-*t* test and the Mann–Whitney *U* test. P values less than 0.05 were considered statistically significant and all tests were two-tailed. Fisher analysis was used to compare the incidences of lymph node metastasis in animal tissues. The isobologram study was used for the determination of synergy as described above.

## Additional file

Additional file 1: Figure S1. Quantitative Data of Westerns Blots (relevant to Fig. 4a, b). **A**. VCaP cells densitomentry data **B**. PC-3 cells densitometry data. **Figure S2** Quantitative data of Western Blots on 2 different CXCR4siRNAs for validation and determination of their effect size (relevant to Fig. 5a). **A**. CXCR4si7 is statistically significant in inhibiting CXCR4 protein expression than NCsi is (*p* < 0.001) and CXCR4si8 is (*p* = 0.002) in VCaP cells. **B**. CXCR4si7 is statistically significant in inhibiting CXCR4 protein expression than NCsi is (*p* < 0.001) and CXCR4si8 is (*p* = 0.01) in PC-3 cells.

## Abbreviations

JNK: c- Jun NH2-terminal kinase
ROS: Reactive oxygen species
AR: Androgen receptor
mCRPC: Metastatic castrate resistant prostate cancer
GLIPR1: Gene encoding the human glioma pathogenesis-related protein 1
ERK1/2: Extracellular signal-regulated kinase1/2
CXCR4: C-X-C chemokine receptor type 4
