# Supplementary material for: GLIPR1-ΔTM synergizes with docetaxel in cell death and suppresses resistance to docetaxel in prostate cancer cells
Source: Mol Cancer. 2015 Jun 19;14:122. doi: 10.1186/s12943-015-0395-0 (PMC4484888; doi:10.1186/s12943-015-0395-0)
Supplement: Additional file 1: Figure S1. — Quantitative Data of Westerns Blots (relevant to Fig. 4a, b). A. VCaP cells densitomentry data B. PC-3 cells densitometry data. Figure S2 Quantitative data of Western Blots on 2 different CXCR4siRNAs for validation and determination of their effect size (relevant to Fig. 5a). A. CXCR4si7 is statistically significant in inhibiting CXCR4 protein expression than NCsi is (p < 0.001) and CXCR4si8 is (p = 0.002) in VCaP cells. B. CXCR4si7 is statistically significant in inhibiting CXCR4 protein expression than NCsi is (p < 0.001) and CXCR4si8 is (p = 0.01) in PC-3 cells. [file 12943_2015_395_MOESM1_ESM.pptx]

## Slide 1
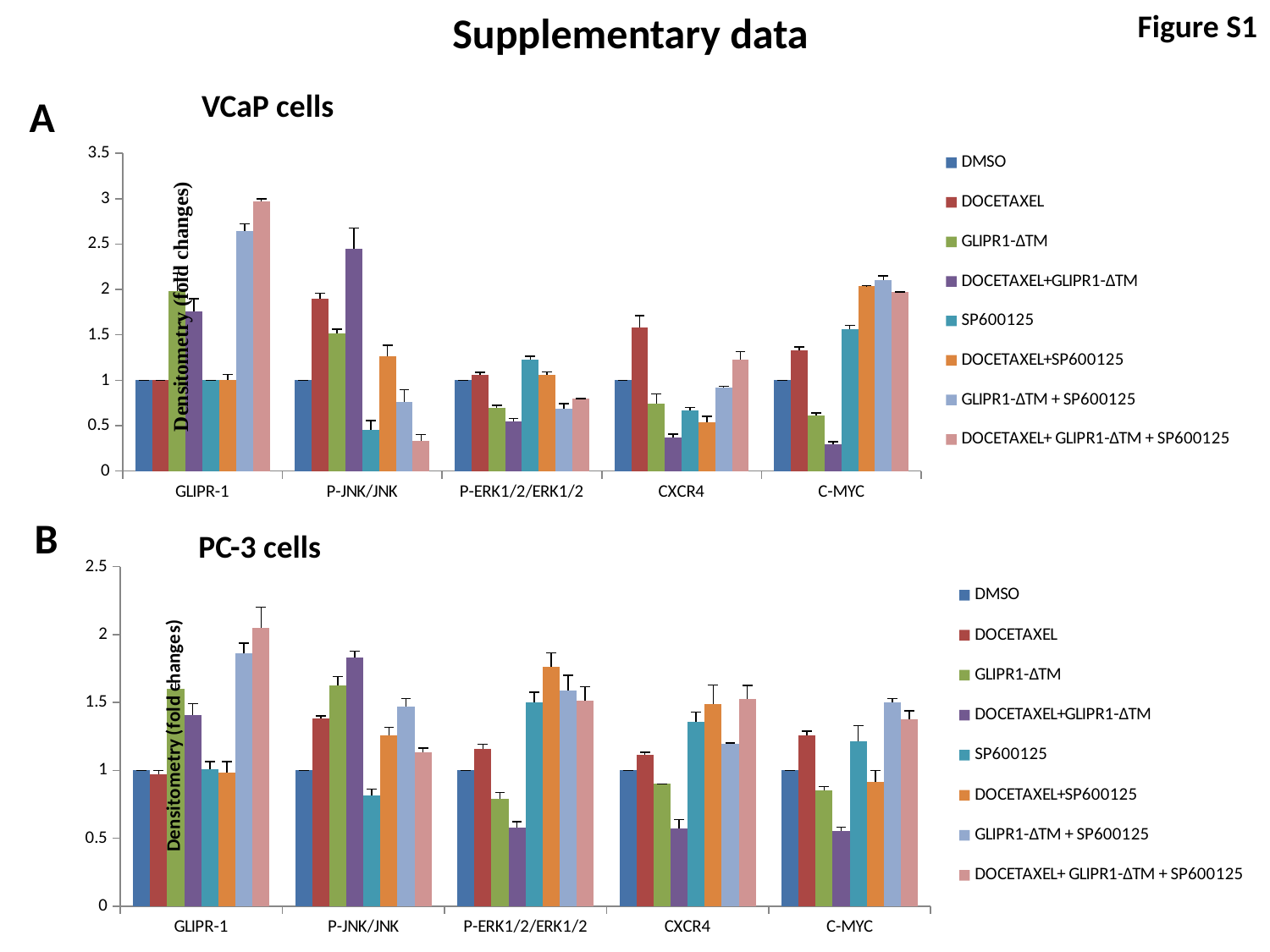

Supplementary data
Figure S1
VCaP cells
A
### Chart
| Category | DMSO | DOCETAXEL | GLIPR1-ΔTM | DOCETAXEL+GLIPR1-ΔTM | SP600125 | DOCETAXEL+SP600125 | GLIPR1-ΔTM + SP600125 | DOCETAXEL+ GLIPR1-ΔTM + SP600125 |
|---|---|---|---|---|---|---|---|---|
| GLIPR-1 | 1.0 | 1.0 | 1.9843137254901961 | 1.7549019607843137 | 1.0 | 1.0067340067340067 | 2.642105263157895 | 2.966666666666667 |
| P-JNK/JNK | 1.0 | 1.9000000000000001 | 1.5148148148148148 | 2.444444444444444 | 0.45303030303030295 | 1.261111111111111 | 0.762962962962963 | 0.33 |
| P-ERK1/2/ERK1/2 | 1.0 | 1.056644880174292 | 0.6975748194014448 | 0.55 | 1.2272727272727273 | 1.0593739250086 | 0.6900584795321638 | 0.7964912280701754 |
| CXCR4 | 1.0 | 1.5833333333333333 | 0.7470588235294118 | 0.3657952069716776 | 0.6666666666666666 | 0.5404040404040403 | 0.9157894736842106 | 1.2296296296296296 |
| C-MYC | 1.0 | 1.3333333333333333 | 0.6156862745098038 | 0.2954248366013072 | 1.5666666666666667 | 2.0377104377104374 | 2.1052631578947367 | 1.9703703703703705 |B
PC-3 cells
### Chart
| Category | DMSO | DOCETAXEL | GLIPR1-ΔTM | DOCETAXEL+GLIPR1-ΔTM | SP600125 | DOCETAXEL+SP600125 | GLIPR1-ΔTM + SP600125 | DOCETAXEL+ GLIPR1-ΔTM + SP600125 |
|---|---|---|---|---|---|---|---|---|
| GLIPR-1 | 1.0 | 0.9696969696969697 | 1.5999999999999999 | 1.409427609427609 | 1.0067340067340067 | 0.9814814814814815 | 1.8590909090909091 | 2.0500000000000003 |
| P-JNK/JNK | 1.0 | 1.383333333333333 | 1.6261261261261264 | 1.830808080808081 | 0.8138998682476943 | 1.2601010101010102 | 1.471212121212121 | 1.1338383838383839 |
| P-ERK1/2/ERK1/2 | 1.0 | 1.1606060606060604 | 0.7878388278388279 | 0.5801282051282052 | 1.503030303030303 | 1.7648148148148148 | 1.587878787878788 | 1.5105401844532278 |
| CXCR4 | 1.0 | 1.1136363636363635 | 0.9 | 0.5737373737373738 | 1.3575757575757574 | 1.4907407407407407 | 1.193939393939394 | 1.527777777777778 |
| C-MYC | 1.0 | 1.2575757575757576 | 0.8500000000000001 | 0.5518518518518518 | 1.2117845117845116 | 0.9166666666666666 | 1.5015151515151517 | 1.377777777777778 |

## Slide 2
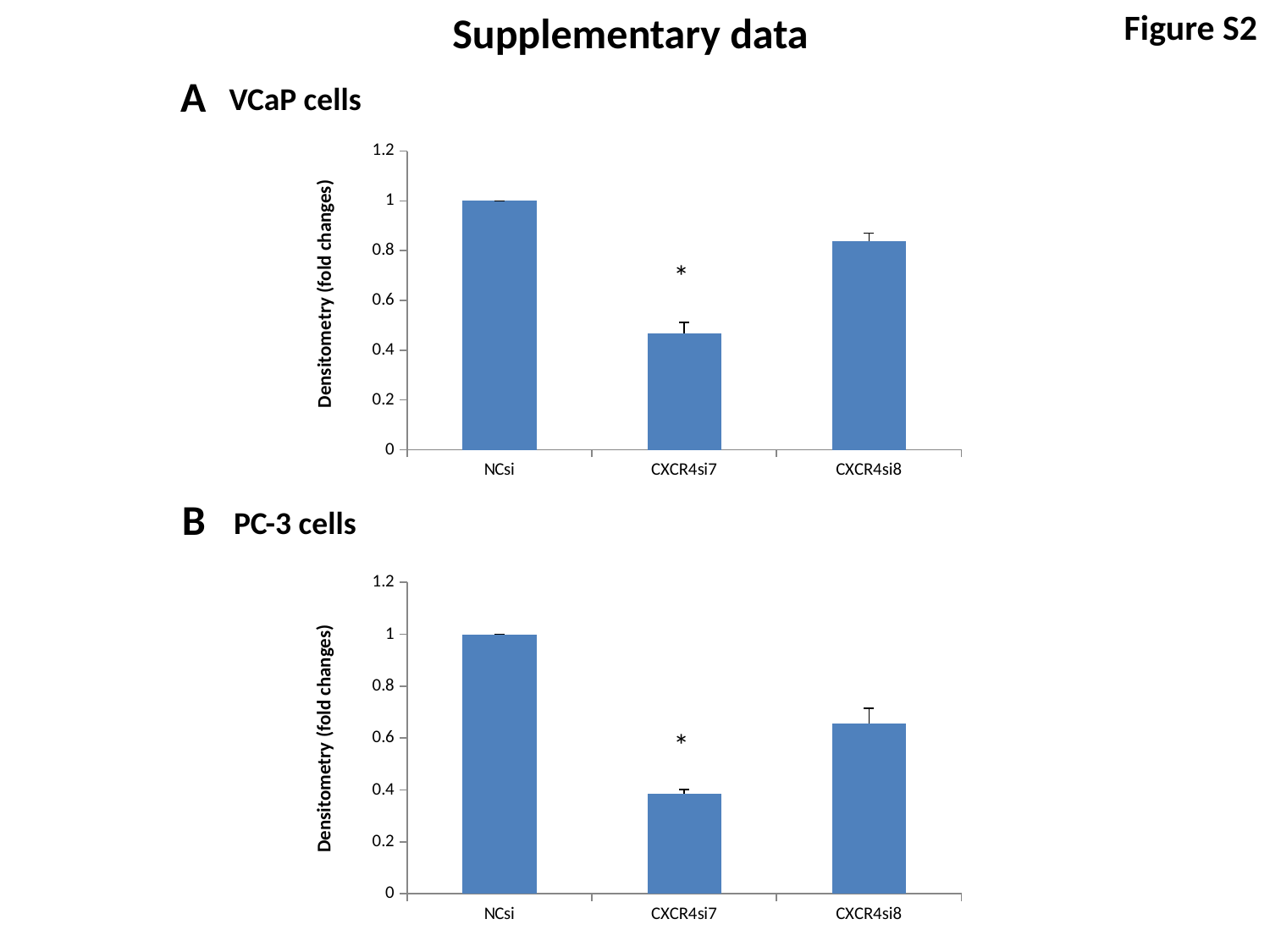

Supplementary data
Figure S2
A
VCaP cells
### Chart
| Category | CXCR4 |
|---|---|
| NCsi | 1.0 |
| CXCR4si7 | 0.4681818181818182 |
| CXCR4si8 | 0.8393939393939395 |Densitometry (fold changes)
*
B
PC-3 cells
### Chart
| Category | CXCR4 |
|---|---|
| NCsi | 1.0 |
| CXCR4si7 | 0.3851010101010101 |
| CXCR4si8 | 0.6563131313131313 |Densitometry (fold changes)
*
